# Results from the centers for disease control and prevention’s predict the 2013–2014 Influenza Season Challenge

**DOI:** 10.1186/s12879-016-1669-x

**Published:** 2016-07-22

**Authors:** Matthew Biggerstaff, David Alper, Mark Dredze, Spencer Fox, Isaac Chun-Hai Fung, Kyle S. Hickmann, Bryan Lewis, Roni Rosenfeld, Jeffrey Shaman, Ming-Hsiang Tsou, Paola Velardi, Alessandro Vespignani, Lyn Finelli, for the Influenza Forecasting Contest Working Group

**Affiliations:** Epidemiology and Prevention Branch, Influenza Division, Centers for Disease Control and Prevention, Atlanta, Georgia USA; Everyday Health, New York, New York USA; Johns Hopkins University, Baltimore, Maryland USA; University of Texas at Austin, Austin, Texas USA; Georgia Southern University, Statesboro, Georgia USA; Los Alamos National Laboratory, Los Alamos, New Mexico USA; Tulane University, New Orleans, Louisiana USA; Virginia Tech, Blacksburg, Virginia USA; Carnegie Mellon University, Pittsburgh, Pennsylvania USA; Columbia University, New York, New York USA; San Diego State University, San Diego, California USA; Sapienza University of Roma, Rome, Italy; Northeastern University, Boston, Massachusetts USA

**Keywords:** Influenza, Forecasting, Prediction, Modeling

## Abstract

**Background:**

Early insights into the timing of the start, peak, and intensity of the influenza season could be useful in planning influenza prevention and control activities. To encourage development and innovation in influenza forecasting, the Centers for Disease Control and Prevention (CDC) organized a challenge to predict the 2013–14 Unites States influenza season.

**Methods:**

Challenge contestants were asked to forecast the start, peak, and intensity of the 2013–2014 influenza season at the national level and at any or all Health and Human Services (HHS) region level(s). The challenge ran from December 1, 2013–March 27, 2014; contestants were required to submit 9 biweekly forecasts at the national level to be eligible. The selection of the winner was based on expert evaluation of the methodology used to make the prediction and the accuracy of the prediction as judged against the U.S. Outpatient Influenza-like Illness Surveillance Network (ILINet).

**Results:**

Nine teams submitted 13 forecasts for all required milestones. The first forecast was due on December 2, 2013; 3/13 forecasts received correctly predicted the start of the influenza season within one week, 1/13 predicted the peak within 1 week, 3/13 predicted the peak ILINet percentage within 1 %, and 4/13 predicted the season duration within 1 week. For the prediction due on December 19, 2013, the number of forecasts that correctly forecasted the peak week increased to 2/13, the peak percentage to 6/13, and the duration of the season to 6/13. As the season progressed, the forecasts became more stable and were closer to the season milestones.

**Conclusion:**

Forecasting has become technically feasible, but further efforts are needed to improve forecast accuracy so that policy makers can reliably use these predictions. CDC and challenge contestants plan to build upon the methods developed during this contest to improve the accuracy of influenza forecasts.

**Electronic supplementary material:**

The online version of this article (doi:10.1186/s12879-016-1669-x) contains supplementary material, which is available to authorized users.

## Background

Each year annual seasonal epidemics of influenza occur in the United States; however, these seasonal epidemics vary in their timing and intensity [1–3]. Preparing for and responding appropriately to influenza epidemics and pandemics is a critical function of public health. Traditionally, planning and response have relied on surveillance data to provide situational awareness [3–5] and information on historic experiences to inform qualitative judgments about what may happen next. A promising new approach has emerged that could help provide a timelier and systematic foundation for public health decision-making: infectious disease forecasting. Infectious disease forecasting combines traditional and internet-derived data on influenza activity with novel mathematical strategies to forecast the progression of an epidemic over a season. These forecasts can provide information for early public health action, such as targeting resources for influenza prevention and control and communicating prevention messages to the public. Initial work describing influenza forecasting methods was promising [6, 7].

To better understand influenza forecasts and improve their usefulness to public health decision making, the Centers for Disease Control and Prevention (CDC) organized a flu forecasting challenge with the primary objectives of 1) examining the accuracy of influenza forecasts, 2) increasing interest in influenza forecasting, and 3) improving the utility of influenza forecasts. CDC also wanted to encourage researchers to utilize novel sources of digital surveillance data (e.g., Twitter data, internet search query data, internet-based surveys) to make their forecasts and connect forecasts to public health decision making. The development of better forecasting models would help CDC improve its ability to monitor influenza in the United States and ultimately improve the prevention and control of influenza.

On November 25, 2013, CDC announced the Predict the Influenza Season Challenge [8]. Challenge contestants were asked to forecast the start, peak, and intensity of the 2013–2014 influenza season at the national level and at any or all Health and Human Services (HHS) region level(s) in the United States. Contestants were free to use any mathematical or statistical model that utilized digital surveillance data. In this report, we present the aggregated results and the lessons learned from the challenge.

## Methods

The requirements and criteria for the selection of the winner have been described more fully in the Federal Register Notice announcing the challenge on November 23, 2013 [8]. Briefly, the challenge period ran from December 1, 2013–March 27, 2014, and contestants were required to submit a total of 9 biweekly forecasts over the challenge period that contained forecasts for the start, peak, length, and intensity of the 2013–2014 influenza season at the national level to be eligible for judging. Teams had to use a form of digital surveillance data (e.g., Twitter data, mining internet search term data, Internet-based surveys) as part of their forecasts but could use other data sources, including those of traditional influenza surveillance systems (e.g. the U.S. Outpatient Influenza-like Illness Surveillance Network [ILINet]). A team’s submission also had to include a narrative describing the methodology of the forecasting model. The forecasting methodology could be changed during the course of the contest, but teams had to submit an updated narrative describing the changes.

All milestones were compared to ILINet. The current ILINet system began during the 1997–1998 influenza season, and ILINet has since demonstrated the ability to provide accurate information on the timing and impact of influenza activity each season [9]. ILINet consists of more than 2,900 outpatient healthcare providers around the country who report data to CDC weekly on the number of patients with influenza-like illness (ILI) and the total number of patients seen in their practices [5]. ILINet data are based on a reporting week that starts on Sunday and ends on Saturday of each week; data are reported through the FluView surveillance report the following Friday [4]. Therefore, the most current ILINet data can lag the calendar date by 1–2 weeks.

We defined the start of the season as the first surveillance week in ILINet where the weighted number of visits for ILI divided by the total number of patient visits (the ILINet percentage) was above the national baseline value of 2.0 % and remained there for at least two additional weeks. We defined the peak week of the season as the surveillance week that the ILINet percentage was the highest. Two values were used to measure the intensity of the influenza season. The first was season duration, which was defined as the number of weeks that the ILINet percentage remained above baseline. The second was the highest numeric value that the ILINet percentage reached in the United States [4]. Weeks for the contest were defined by Morbidity and Mortality Weekly Report (MMWR) surveillance weeks; the MMWR calendar is available at http://wwwn.cdc.gov/nndss/script/downloads.aspx. Contestants were also eligible to submit milestone forecasts for any or all of the 10 HHS regions to add to their final scores.

Forecasts were considered accurate if they were within 1 week or one percent of the actual value calculated from ILINet. The selection of the winner for this challenge was based on an evaluation of the methodology used to make the forecast and the accuracy of the forecast. Contestant submissions were judged by a panel of reviewers that included two CDC staff outside the Influenza Division and one faculty member from a noncompeting university. Judges scored submissions on a scale of 0 to 100 points using the following criteria: the strength of the methodology (25 points), which assessed how clearly the results and uncertainty in the forecasts were presented and how the data sources and forecast methodology were described; the accuracy, timeliness, and reliability of the forecasts for the start, peak week, and intensity of the influenza season (65 points); and the scope of the geography (US plus one or more HHS Regions) that the source data represented (10 points). Up to 50 bonus points were awarded to any contestant that submitted forecasts for the 10 HHS regions; the number of bonus points was based on the number of regions with a forecast and the strength of the methodology and the accuracy, timeliness, and reliability of the forecasts. The winner was awarded a cash prize of $75,000. The results presented in this analysis are based on the forecasts from teams that met the eligibility criteria.

## Results

Sixteen individuals or teams initially registered for the challenge, 15 entered at least one forecast, 11 submitted nine biweekly forecasts, and 9 submitted forecasts for all required milestones and are included in this report. The majority of teams used Twitter (*n* = 6 teams) and/or Google Flu Trends data (*n* = 5 teams) as a data source to inform their forecasting models. Teams also utilized digital data sources such as Wikipedia search inquiries and HealthMap data; 3/9 (33 %) teams utilized more than one digital data source (Table 1). Five out of 9 (56 %) teams employed statistical methods like time series analysis and generalized linear models, and 4/9 (44 %) employed mechanistic models that incorporated compartmental modeling (e.g., Susceptible-Exposed-Infected-Recovered [SEIR] models) (Table 1). Eight out of 9 teams made forecasts for at least one HHS region during the challenge period (Table 1). Two teams provided multiple forecasts using distinct methods or data sources as part of their submission. A total of 13 forecasts were evaluated over the contest period.

**Table 1:** Characteristics of nine teams that competed in the Predict the 2013–14 Influenza Season Challenge

Team	Digital Data source	Model type	Regional forecast^a^	Brief description^d^
A	Wikipedia	mechanistic^b^	Yes	Susceptible-Exposed-Infected-Recovered (SEIR) model using data assimilation to probabilistically fit models to ILINet data
B	Twitter	mechanistic	Yes	SEIR model initialized with current Twitter and ILINet data
C	Google Flu Trends; Twitter	statistical^c^	Yes	Utilized method of analogues, Kalman filtering, Poisson regression, and an ensemble method averaging the results of the three models to forecast ILINet
D	Google Flu Trends	statistical	Yes	Utilized empirical Bayes model and a spatio-temporal likelihood function
E	Google Flu Trends; Twitter	statistical	Yes	Utilized multiplicative time series model
F	Google Flu Trends	mechanistic	Yes	Susceptible-infected-recovered-susceptible (SIRS) model initialized with Google Flu Trends data and data assimilation methods
G	Twitter	statistical	No	Extrapolation of filtered Twitter data
H	Google Flu Trends; HealthMap; Twitter	mechanistic	Yes	Statistical models used to make short term forecasts and agent based models combined with mean field models with non-linear optimization techniques used to output long term forecasts.
I	Twitter	statistical	Yes	Utilized time series model and method of analogues

^a^Yes denotes forecast for ≥1 region (for all weeks)

^b^Includes models that incorporate compartmental modeling like Susceptible-Exposed-Infected-Recovered [SEIR] models

^c^Includes models like time series analysis and generalized linear models

^d^Additional information on methodology and results for select teams available in references [34–38]

**Fig. 1:**
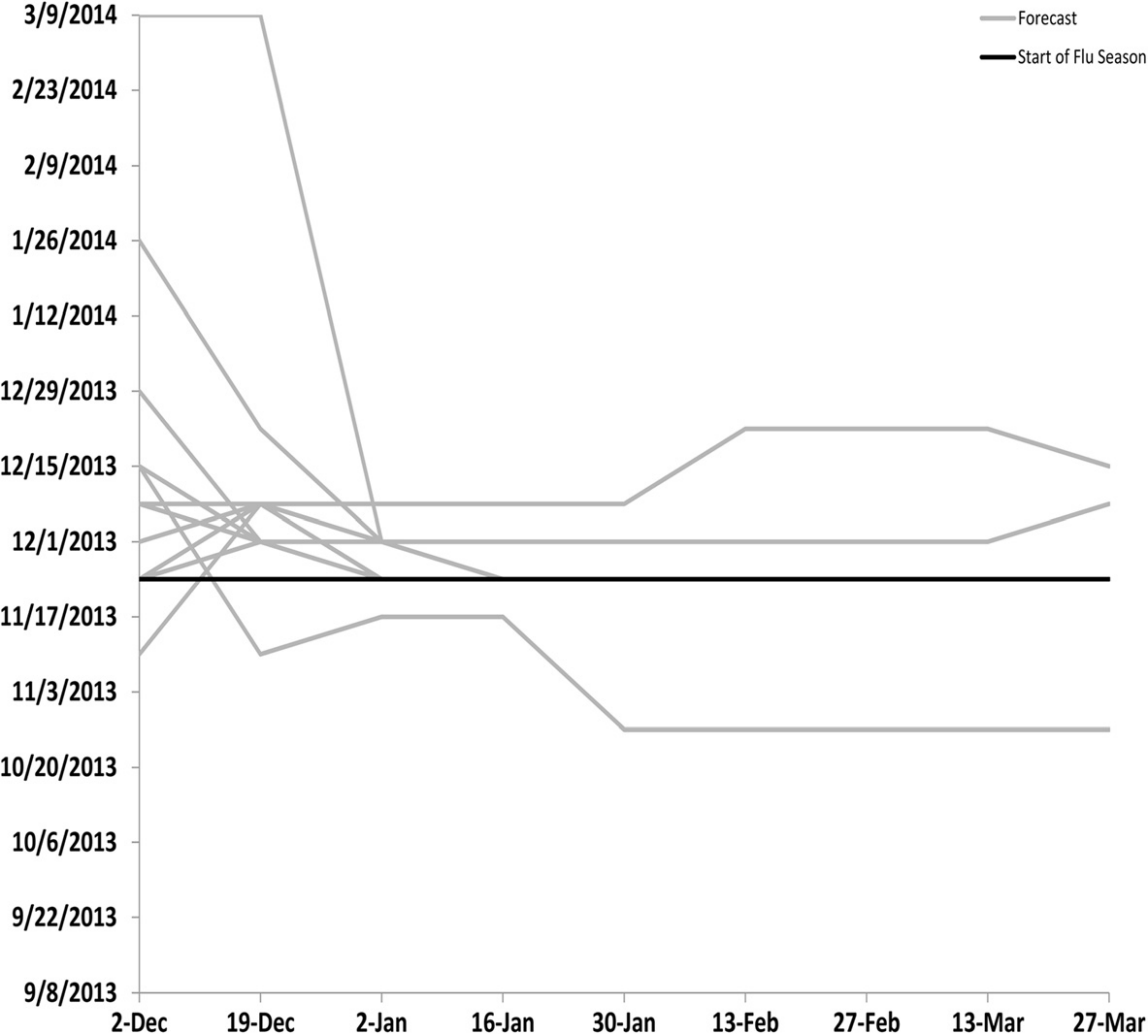
Forecasted start week of the 2013–2014 influenza season, by forecast date, United States (*n* = 13). Forecasts presented here are from teams that successfully completed the CDC Predict the 2013–2014 Influenza Season Challenge. The start of the season was defined as the first surveillance week in ILINet where the number of visits for ILI divided by the total number of patient visits (the ILINet percentage) was above the national baseline value of 2.0 % and remained there for at least two additional weeks

**Fig. 2:**
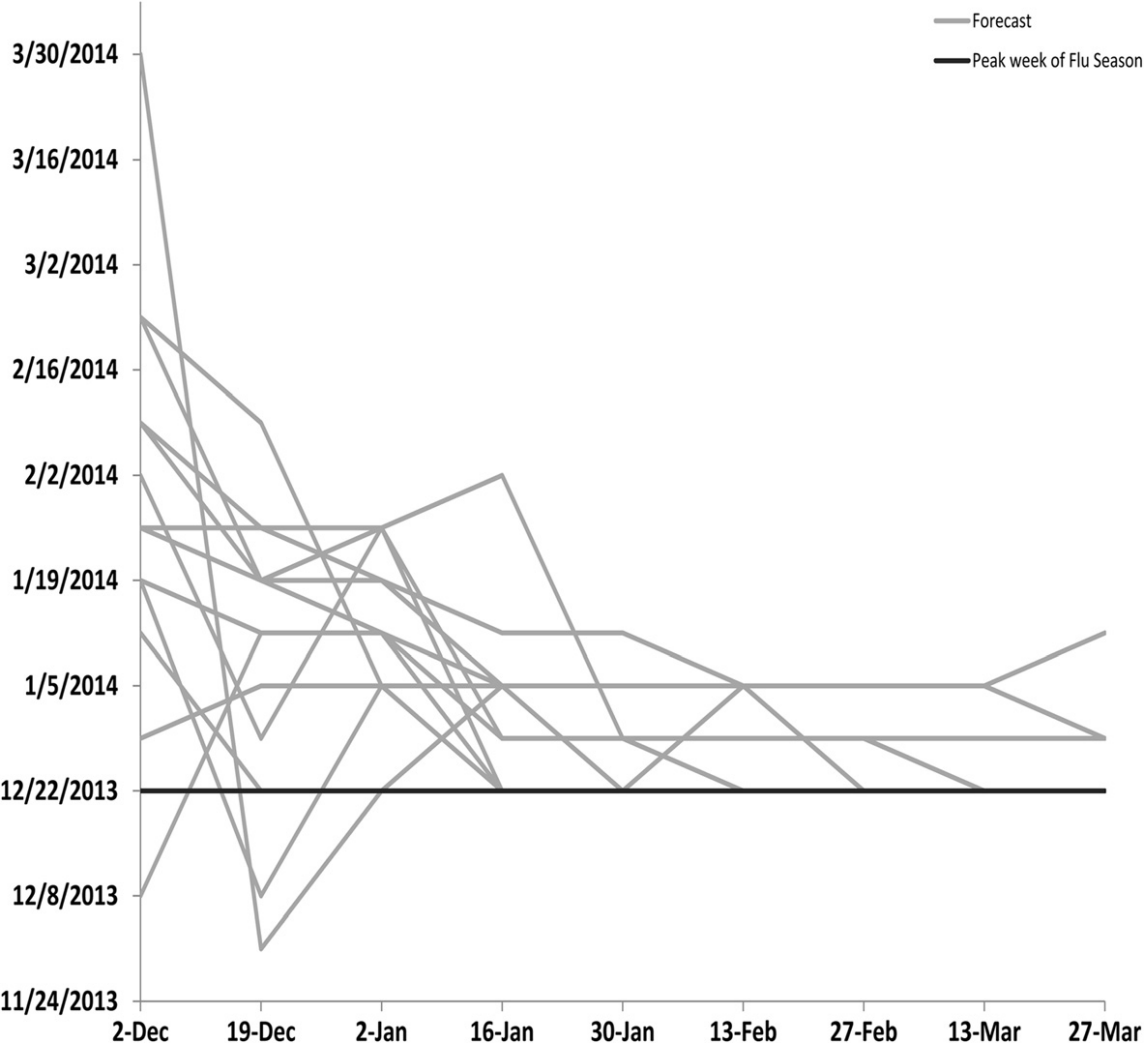
Forecasted peak week of the 2013–2014 influenza season, by forecast date, United States (*n* = 13). Forecasts presented here are from teams that successfully completed the CDC Predict the 2013–2014 Influenza Season Challenge. The peak week of the season was defined as the surveillance week that the ILINet percentage was the highest during the 2013–14 influenza season

**Fig. 3:**
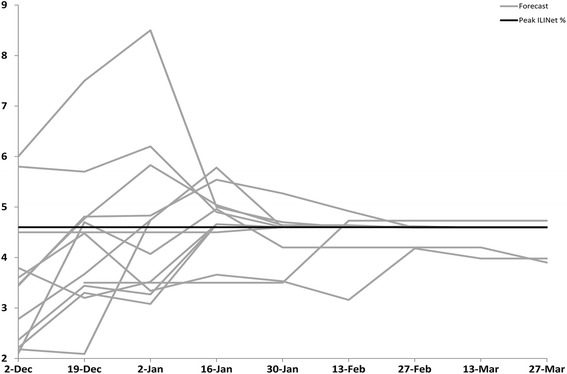
Forecasted peak ILINet percent of the 2013–2014 influenza season, by forecast date, United States (*n* = 13). Forecasts presented here are from teams that successfully completed the CDC Predict the 2013–2014 Influenza Season Challenge. The ILINet percent peak was defined as the highest numeric value that the ILINet percentage reached in the United States during the 2013–14 influenza season

**Fig. 4:**
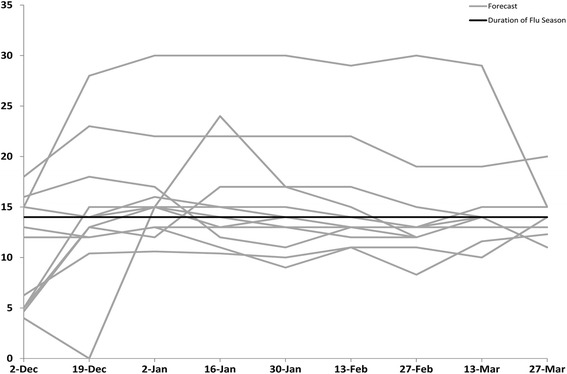
Forecasted duration of the 2013–2014 influenza season, by forecast date, United States (*n* = 13). Forecasts presented here are from teams that successfully completed the CDC Predict the 2013–2014 Influenza Season Challenge. The duration was defined as the number of weeks that the ILINet percentage remained above the national baseline

Based on values from ILINet, the 2013–14 influenza season in the United States began on MMWR week 48 (late November), peaked on week 52 (late December) at 4.6 %, and lasted for 14 consecutive weeks (Table 2). Virologic data indicated that pH1N1 viruses predominated nationally and in all 10 regions for the majority of the influenza season but that influenza B viruses became the predominant virus nationally in week 13 (late March) [3]. The median submitted milestone forecasts are shown for the United States in Table 3 and for the 10 HHS regions in Additional file 1: Tables S1–S10. The 13 biweekly forecasts of the milestones for the United States submitted by the 9 teams (the grey lines) are presented in Figs. 1, 2, 3 and 4 along with the milestones as calculated from ILINet (the black line). The first forecast was due on December 2, 2013, when ILINet data for surveillance week 46 were available. The median value of the 13 forecasts received for the start of the influenza season was week 50 (corresponding to the calendar week beginning December 8, 2013), for the week that the ILINet percentage would peak was week 5 (the week beginning January 26, 2014), for the ILINet peak was 3.45 %, and for the median forecasted season duration was 13 weeks (Table 3). When compared to the ILINet results, 3/13 (23 %) individual forecasts correctly forecasted the start of the influenza season within one week, 1/13 (8 %) correctly forecasted the peak within 1 week, 3/13 (23 %) correctly forecasted the peak ILINet percentage within 1 %, and 4/13 (31 %) correctly forecasted the duration within 1 week (Table 4); national-level accuracy results for the 13 predictions received by each milestone are available in Additional file 1: Tables S11–S14.

**Table 2:** Forecasting targets for the 2013–2014 influenza season as calculated from the U.S. Outpatient Influenza-like Illness Surveillance Network (ILINet), United States

	Start week^a^	Peak week^a^	Peak percentage	Duration of influenza season
United States	48	52	4.6	14

*Legend:* The start of the season was defined as the first surveillance week in ILINet where the number of visits for ILI divided by the total number of patient visits (the ILINet percentage) was above the national baseline value of 2.0 % and remained there for at least two additional weeks. The peak week of the season was defined as the surveillance week that the ILINet percentage was the highest during the 2013–14 influenza season. The ILINet percent peak was defined as the highest numeric value that the ILINet percentage reached in the United States during the 2013–14 influenza season. The duration was defined as the number of weeks that the ILINet percentage remained above the national baseline.

^a^Weeks are given in Morbidity and Mortality Weekly Report surveillance weeks. For calendar start and end dates of each week, please see http://wwwn.cdc.gov/nndss/script/downloads.aspx

**Table 3:** Median forecasted start week, peak week, peak percentage, and duration of the 2013–14 influenza season, by forecast date, United States (*n* = 13)

Date of forecast (Week of ILINet data availability^a,b^)	Median forecasted start week^b^	Median forecasted peak week^b^	Median forecasted peak percentage	Median forecasted duration of influenza season
12/2/2013 (WK. 46)	50	5	3.5	13
12/19/2013 (WK. 49)	49	3	4.5	14
1/2/2014 (WK. 51)	48	3	4.5	15
1/16/2014 (WK.1)	48	2	4.9	14
1/30/2014 (WK. 3)	48	52	4.6	14
2/13/2014 (WK. 5)	48	52	4.6	13
2/27/2014 (WK. 7)	48	52	4.6	13
3/13/2014 (WK. 9)	48	52	4.6	14
3/27/2014 (WK. 11)	48	52	4.6	14

*Legend:* Forecasts presented here are from the 9 teams that successfully completed the CDC Predict the 2013–2014 Influenza Season Challenge

^a^ILINet data are based on a reporting week that starts on Sunday and ends on Saturday of each week, and data are reported out through the FluView surveillance report the following Friday. Therefore, the most current ILINet data can lag the calendar date by 1–2 weeks

^b^Weeks are given in Morbidity and Mortality Weekly Report surveillance weeks. For calendar start and end dates of each week, please see http://wwwn.cdc.gov/nndss/script/downloads.aspx

**Table 4:** Forecasts within 1 week or percent of the start week, peak week, peak percentage, and duration of the 2013–14 influenza season, by forecast date, United States (n = 13)

Date of forecast (Week of ILINet data availability^a,b^)	Start week	Peak week	Peak percentage	Duration of influenza season
12/2/2013 (WK. 46)	3 (23 %)	1 (8 %)	3 (23 %)	4 (31 %)
12/19/2013 (WK. 49)	6 (46 %)	2 (15 %)	6 (46 %)	6 (46 %)
1/2/2014 (WK. 51)	12 (92 %)^c^	2 (15 %)	5 (38 %)	7 (54 %)
1/16/2014 (WK.1)	12 (92 %)	6 (46 %)	10 (77 %)	6 (46 %)
1/30/2014 (WK. 3)	11 (85 %)	11 (85 %)	11 (85 %)	6 (46 %)
2/13/2014 (WK. 5)	11 (85 %)	10 (77 %)	12 (92 %)	6 (46 %)
2/27/2014 (WK. 7)	11 (85 %)	11 (85 %)	13 (100 %)	5 (38 %)
3/13/2014 (WK. 9)	11 (85 %)	11 (85 %)	13 (100 %)	9 (69 %)
3/27/2014 (WK. 11)	10 (77 %)	12 (92 %)	13 (100 %)	10 (77 %)

*Legend:* Forecasts presented here are from the 9 teams that successfully completed the CDC Predict the 2013–2014 Influenza Season Challenge. The start of the season was defined as the first surveillance week in ILINet where the number of visits for ILI divided by the total number of patient visits (the ILINet percentage) was above the national baseline value of 2.0 % and remained there for at least two additional weeks. The peak week of the season was defined as the surveillance week that the ILINet percentage was the highest during the 2013–14 influenza season. The ILINet percent peak was defined as the highest numeric value that the ILINet percentage reached in the United States during the 2013–14 influenza season. The duration was defined as the number of weeks that the ILINet percentage remained above the national baseline

^a^ILINet data are based on a reporting week that starts on Sunday and ends on Saturday of each week, and data are reported out through the FluView surveillance report the following Friday. Therefore, the most current ILINet data can lag the calendar date by 1–2 weeks

^b^Weeks are given in Morbidity and Mortality Weekly Report surveillance weeks. For calendar start and end dates of each week, please see http://wwwn.cdc.gov/nndss/script/downloads.aspx

^c^Last forecast received before milestone observed in ILINet

For the forecast submitted on December 19, when ILINet data from surveillance week 49 were available, the contestants adjusted their models based on updated ILINet and digital data and the median forecast for the peak week shifted from week 5 to week 2 (the week beginning January 5, 2014). The median forecast for the peak ILINet percentage increased to 4.48 %, and the median forecast for season duration increased to 14 weeks. The number of submissions that correctly forecasted the peak week increased to 2/13 (15 %), the peak percentage to 6/13 (46 %), and the duration of the season to 6/13 (46 %). As the season progressed, the median forecasts for peak week, peak percentage, and peak duration for the United States became more stable and a majority of the 13 predictions converged on the season milestones calculated from ILINet (Tables 2, 3 and 4; Figs. 1, 2, 3, 4).

After a review of the forecasts and the results from the ILINet system, the judges found that Jeffrey Shaman’s team from Columbia University was the overall winner of CDC’s Predict the Influenza Season Challenge [10].

## Discussion

This challenge represents the first nationally coordinated effort to forecast an influenza season in the United States. CDC organized the challenge to support the continued technical innovation required to forecast the influenza season. The results of this challenge indicate that while forecasting has become technically feasible and reasonably accurate forecasts can be made in the short term, further work refining forecasting methodology and identifying the best uses of forecasts for public health decision making are needed. CDC continues to work with the researchers who participated in the challenge to refine and adapt methodology and determine the best uses of forecasts to inform public health decision making.

This forecasting challenge was useful to CDC in many ways. First, it promoted the development of forecasting models using data readily available through existing surveillance and digital data (e.g. Google Flu Trends) platforms. Second, it encouraged the connection between forecasters, subject matter experts, and public health decision-makers. During the challenge, forecasters needed support understanding the nuances of CDC’s surveillance data while public health decision makers needed support understanding the different digital data sources and forecasting techniques. This challenge also incorporated forecasting milestones that would be most useful to public health decision makers in planning for influenza prevention and control activities, including the start week of the influenza season. This milestone was rarely included in previous efforts to forecast influenza but could inform public health action, including the development of tailored recommendations to the public about vaccination timing and to physicians about influenza antiviral treatment [6, 7].

The results from the challenge indicated that no team was entirely accurate at forecasting all of the influenza season milestones. Public health actions informed by forecasts that later prove to be inaccurate can have negative consequences, including the loss of credibility, wasted and misdirected resources, and, in the worst case, increases in morbidity or mortality. There are at least two possible reasons for the lack of accuracy; the first is the use of digital data. Multiple published scientific studies show that digital surveillance data, such as Twitter, Wikipedia search queries, and Google Flu Trends data, have correlation with influenza activity as measured by existing traditional influenza surveillance programs and have been used successfully to track vaccine sentiment and monitor disease outbreaks [11–26], often in a timelier manner than traditional infectious disease surveillance data. However, not all digital data are equally accurate, and the algorithms and methodologies underpinning these data require constant upkeep to maintain their accuracy [27]. Several contestants in this challenge utilized Google Flu Trends to inform their forecasts, which has been shown to have overestimated influenza activity in recent seasons [28, 29]. Influenza forecasting models informed by digital data are subject to the biases and errors of their underlying source data, and few contestants in this study utilized more than one digital data set, which could make influenza forecasts more robust to data biases.

The second reason that forecasts may not have been accurate relates to the model methodologies and specifications themselves. While substantial work has advanced the field of infectious disease forecasting, it is still in the early years of development. Recent reviews of influenza forecasting found the use of a variety of statistical and infectious disease modeling approaches, including time series models, SEIR models, and agent-based models [6, 7]. While the advantages and limitations of these approaches have been described, the impact on the relative accuracies of these approaches are unknown, and optimal forecasting methods are still under study [7]. The results of this contest, while helpful to gauge the general accuracy of influenza forecasting, cannot be used to recommend the best forecasting methodology because participants in this contest used different data sources and methodological approaches to make their forecasts. Forecasts could have been improved by the use of more precise digital data, better forecasting methodology, or both. Additionally, the challenge ran for only one influenza season. Because of the unpredictable timing and intensity of influenza seasons in the United States, assessments of forecasting methods and accuracy will require a review over multiple influenza seasons to ensure consistency in performance.

This challenge identified a number of barriers limiting forecasting model development and application, adoption by decision-makers, and the eventual public health impact of forecasts. First, interaction between model developers and public health decision-makers has been limited, leading to difficulties in identifying and specifying relevant prediction targets that would best inform decision-making. Second, data for making and evaluating predictions are often difficult to share, obtain, and interpret. Third, no common standards exist for evaluating models, either against each other or against metrics relevant to decision-makers, making it difficult to evaluate the accuracy and reliability of forecasts. Lastly, the presentation of the forecast confidence varied between teams, making comparison and interpretation by decision makers difficult. CDC and challenge contestants continue to work together through collaborative challenges to forecast the 2014–15 and 2015–16 influenza seasons in order to improve data availability and interpretation, develop standardized metrics to assess forecast accuracy and standardized ways to communicate forecasts and their uncertainty, and identify the types of decisions best aided by forecasts [30]. The identified improvements will not only increase the utility of forecasts for public health decision making in influenza but will be relevant to the forecasting of other infectious diseases, which face similar challenges. The best practices and lessons learned from influenza have already been shared with other government-run infectious disease forecasting challenges [31, 32].

The Predict the Influenza Season Challenge represents a successful utilization of the COMPETES Act, which authorized U.S. government agencies to host challenges to improve government and encourage innovation [33]. By hosting this challenge, CDC was able to receive and evaluate influenza season forecasts from 9 teams based on a variety of digital data sources and methodologies [34–38]. These forecasts were submitted by teams that were affiliated with a diverse set of organizations including universities and private industry, and some of the teams had never produced an influenza forecast before participation in the challenge. The high number of forecasts received through this challenge is in contrast to the number of forecasts that would have been received if a more traditional method of outside engagement available at CDC was utilized (e.g., traditional contracts or grants). The challenge mechanism allowed CDC to establish the overall goal of accurately forecasting influenza season milestones without specifying the forecasting methodologies and allowed CDC to evaluate forecasts for accuracy and quality prior to awarding of the challenge prize.

## Conclusions

CDC currently monitors influenza activity each year using routine influenza surveillance systems that do not forecast influenza activity [3]. To help promote the continued advancement of influenza forecasting, CDC held the first nationally coordinated challenge to forecast the influenza season in the United States. Nine teams representing academia, private industry, and public health made forecasts about the start, the duration, and the intensity of the 2013–14 influenza season using a variety of digital data sources and methods. While no team accurately forecasted all influenza season milestones, the results of this challenge indicate that reasonably accurate forecasts can be made in the short term. Further work to refine and adapt forecasting methodology and identify the best uses of forecasts for public health decision making is required before the results of influenza forecasting can be used by policy makers and the public to inform the selection and implementation of prevention and control measures. Nevertheless, forecasting holds much promise for seasonal influenza prevention and control and pandemic preparedness.

## Abbreviations

CDC, centers for disease control and prevention; HHS, health and human services; ILI, influenza-like illness; ILINet, U.S. outpatient influenza-like illness surveillance network; MMWR, morbidity and mortality weekly report; SEIR, susceptible-exposed-infected-recovered; SIRS, susceptible-infected-recovered-susceptible; US, United States

## Additional file

Additional file 1: Median predicted start week, peak week, peak % ILINet, and duration of season for the 10 HHS Regions, and national-level forecasts that were within 1 week or 1 percent of the start week, peak week, peak % ILINet, and duration of season, CDC’s Predict the 2013–2014 Influenza Season Challenge. Tables S1-10 contains the median submitted milestone forecasts and the milestones as calculated from ILINet for the 10 HHS regions (Tables S1–S10). Tables S11–14 contain the national-level accuracy results for the 13 predictions received by each milestone. (DOCX 47 kb)
