# Supplementary material for: Results from the centers for disease control and prevention’s predict the 2013–2014 Influenza Season Challenge
Source: BMC Infect Dis. 2016 Jul 22;16:357. doi: 10.1186/s12879-016-1669-x (PMC4957319; doi:10.1186/s12879-016-1669-x)
Supplement: Additional file 1: — Median predicted start week, peak week, peak % ILINet, and duration of season for the 10 HHS Regions, and national-level forecasts that were within 1 week or 1 percent of the start week, peak week, peak % ILINet, and duration of season, CDC’s Predict the 2013–2014 Influenza Season Challenge. Tables S1-10 contains the median submitted milestone forecasts and the milestones as calculated from ILINet for the 10 HHS regions (Tables S1–S10). Tables S11–14 contain the national-level accuracy results for the 13 predictions received by each milestone. (DOCX 47 kb) [file 12879_2016_1669_MOESM1_ESM.docx]

Supplemental Table 1. A) Median predicted start week, peak week, peak % ILINet, and duration of season submitted by the 9 teams that completed the CDC Predict the 2013–2014 Influenza Season Challenge, by the date of submission, Health and Human Services Region 1.

B) The start week, peak week, peak ILINet percentage, and duration of season for the 2013–2014 influenza season as calculated from the U.S. Outpatient Influenza-like Illness Surveillance Network (ILINet), United States.

A)

| **Date of prediction**  **(Week of ILINet data availability^1,2^)** | **Median predicted start week^2^** | **Median predicted peak week^2^** | **Median predicted peak percentage** | **Median predicted duration of influenza season** |
| --- | --- | --- | --- | --- |
| 12/2/2013 (WK. 49) | 1 | 7 | 1.6 | 11 |
| 12/19/2013 (WK. 51) | 1 | 6 | 2.4 | 8 |
| 1/2/2014 (WK. 1) | 52 | 5 | 2.5 | 10 |
| 1/16/2014 (WK.3) | 52 | 5 | 3.0 | 12 |
| 1/30/2014 (WK. 5) | 52 | 5 | 2.8 | 11 |
| 2/13/2014 (WK. 7) | 52 | 5 | 2.1 | 10 |
| 2/27/2014 (WK. 9) | 52 | 6 | 2.3 | 11 |
| 3/13/2014 (WK. 11) | 52 | 6 | 2.3 | 9 |
| 3/27/2014 (WK. 13) | 52 | 6 | 2.3 | 12 |

B)

|  | **Start week^2^** | **Peak week^2^** | **Peak percentage** | **Duration of influenza season** |
| --- | --- | --- | --- | --- |
| HHS Region 1 | 52 | 6 | 2.2 | 20 |

**^1^**ILINet data are based on a reporting week that starts on Sunday and ends on Saturday of each week, and data are reported out through the FluView surveillance report the following Friday. Therefore, the most current ILINet data can lag the calendar date by 1–2 weeks.

**^2^**Weeks are given in Morbidity and Mortality Weekly Report surveillance weeks. For calendar start and end dates of each week, please see <http://wwwn.cdc.gov/nndss/script/downloads.aspx>.

Supplemental Table 2. A) Median predicted start week, peak week, peak % ILINet, and duration of season submitted by the 9 teams that completed the CDC Predict the 2013–2014 Influenza Season Challenge, by the date of submission, Health and Human Services Region 2.

B) The start week, peak week, peak ILINet percentage, and duration of season for the 2013–2014 influenza season as calculated from the U.S. Outpatient Influenza-like Illness Surveillance Network (ILINet), United States.

A)

| **Date of prediction**  **(Week of ILINet data availability^1,2^)** | **Median predicted start week^2^** | **Median predicted peak week^2^** | **Median predicted peak percentage** | **Median predicted duration of influenza season** |
| --- | --- | --- | --- | --- |
| 12/2/2013 (WK. 49) | 51 | 5 | 2.6 | 3 |
| 12/19/2013 (WK. 51) | 50 | 2 | 4.4 | 9 |
| 1/2/2014 (WK. 1) | 52 | 3 | 3.7 | 8 |
| 1/16/2014 (WK.3) | 51 | 2 | 3.7 | 9 |
| 1/30/2014 (WK. 5) | 51 | 3 | 3.5 | 10 |
| 2/13/2014 (WK. 7) | 51 | 5 | 3.6 | 11 |
| 2/27/2014 (WK. 9) | 51 | 6 | 3.7 | 12 |
| 3/13/2014 (WK. 11) | 51 | 6 | 3.7 | 10 |
| 3/27/2014 (WK. 13) | 51 | 6 | 3.5 | 13 |

B)

|  | **Start week^2^** | **Peak week^2^** | **Peak percentage** | **Duration of influenza season** |
| --- | --- | --- | --- | --- |
| HHS Region 2 | 51 | 6 | 3.4 | 23 |

**^1^**ILINet data are based on a reporting week that starts on Sunday and ends on Saturday of each week, and data are reported out through the FluView surveillance report the following Friday. Therefore, the most current ILINet data can lag the calendar date by 1–2 weeks.

**^2^**Weeks are given in Morbidity and Mortality Weekly Report surveillance weeks. For calendar start and end dates of each week, please see <http://wwwn.cdc.gov/nndss/script/downloads.aspx>.

Supplemental Table 3. A) Median predicted start week, peak week, peak % ILINet, and duration of season submitted by the 9 teams that completed the CDC Predict the 2013–2014 Influenza Season Challenge, by the date of submission, Health and Human Services Region 3.

B) The start week, peak week, peak ILINet percentage, and duration of season for the 2013–2014 influenza season as calculated from the U.S. Outpatient Influenza-like Illness Surveillance Network (ILINet), United States.

A)

| **Date of prediction**  **(Week of ILINet data availability^1,2^)** | **Median predicted start week^2^** | **Median predicted peak week^2^** | **Median predicted peak percentage** | **Median predicted duration of influenza season** |
| --- | --- | --- | --- | --- |
| 12/2/2013 (WK. 49) | 1 | 6 | 2.4 | 5 |
| 12/19/2013 (WK. 51) | 51 | 5 | 4.3 | 11 |
| 1/2/2014 (WK. 1) | 51 | 3 | 4.5 | 12 |
| 1/16/2014 (WK.3) | 51 | 3 | 4.5 | 15 |
| 1/30/2014 (WK. 5) | 51 | 3 | 3.9 | 13 |
| 2/13/2014 (WK. 7) | 51 | 2 | 3.7 | 13 |
| 2/27/2014 (WK. 9) | 51 | 1 | 3.6 | 11 |
| 3/13/2014 (WK. 11) | 51 | 1 | 3.6 | 10 |
| 3/27/2014 (WK. 13) | 51 | 1 | 3.6 | 10 |

B)

|  | **Start week^2^** | **Peak week^2^** | **Peak percentage** | **Duration of influenza season** |
| --- | --- | --- | --- | --- |
| HHS Region 3 | 51 | 1 | 3.6 | 10 |

**^1^**ILINet data are based on a reporting week that starts on Sunday and ends on Saturday of each week, and data are reported out through the FluView surveillance report the following Friday. Therefore, the most current ILINet data can lag the calendar date by 1–2 weeks.

**^2^**Weeks are given in Morbidity and Mortality Weekly Report surveillance weeks. For calendar start and end dates of each week, please see <http://wwwn.cdc.gov/nndss/script/downloads.aspx>.

Supplemental Table 4. A) Median predicted start week, peak week, peak % ILINet, and duration of season submitted by the 9 teams that completed the CDC Predict the 2013–2014 Influenza Season Challenge, by the date of submission, Health and Human Services Region 4.

B) The start week, peak week, peak ILINet percentage, and duration of season for the 2013–2014 influenza season as calculated from the U.S. Outpatient Influenza-like Illness Surveillance Network (ILINet), United States.

A)

| **Date of prediction**  **(Week of ILINet data availability^1,2^)** | **Median predicted start week^2^** | **Median predicted peak week^2^** | **Median predicted peak percentage** | **Median predicted duration of influenza season** |
| --- | --- | --- | --- | --- |
| 12/2/2013 (WK. 49) | 50 | 6 | 3.9 | 14 |
| 12/19/2013 (WK. 51) | 48 | 1 | 3.9 | 14 |
| 1/2/2014 (WK. 1) | 48 | 2 | 4.7 | 14 |
| 1/16/2014 (WK.3) | 48 | 2 | 4.8 | 14 |
| 1/30/2014 (WK. 5) | 48 | 52 | 4.6 | 12 |
| 2/13/2014 (WK. 7) | 48 | 52 | 4.7 | 12 |
| 2/27/2014 (WK. 9) | 48 | 52 | 4.7 | 12 |
| 3/13/2014 (WK. 11) | 48 | 52 | 4.7 | 12 |
| 3/27/2014 (WK. 13) | 48 | 52 | 4.7 | 12 |

B)

|  | **Start week^2^** | **Peak week^2^** | **Peak percentage** | **Duration of influenza season** |
| --- | --- | --- | --- | --- |
| HHS Region 4 | 48 | 52 | 4.6 | 12 |

**^1^**ILINet data are based on a reporting week that starts on Sunday and ends on Saturday of each week, and data are reported out through the FluView surveillance report the following Friday. Therefore, the most current ILINet data can lag the calendar date by 1–2 weeks.

**^2^**Weeks are given in Morbidity and Mortality Weekly Report surveillance weeks. For calendar start and end dates of each week, please see <http://wwwn.cdc.gov/nndss/script/downloads.aspx>.

Supplemental Table 5. A) Median predicted start week, peak week, peak % ILINet, and duration of season submitted by the 9 teams that completed the CDC Predict the 2013–2014 Influenza Season Challenge, by the date of submission, Health and Human Services Region 5.

B) The start week, peak week, peak ILINet percentage, and duration of season for the 2013–2014 influenza season as calculated from the U.S. Outpatient Influenza-like Illness Surveillance Network (ILINet), United States.

A)

| **Date of prediction**  **(Week of ILINet data availability^1,2^)** | **Median predicted start week^2^** | **Median predicted peak week^2^** | **Median predicted peak percentage** | **Median predicted duration of influenza season** |
| --- | --- | --- | --- | --- |
| 12/2/2013 (WK. 49) | 52 | 7 | 2.8 | 9 |
| 12/19/2013 (WK. 51) | 51 | 6 | 4.2 | 14 |
| 1/2/2014 (WK. 1) | 51 | 4 | 3.8 | 13 |
| 1/16/2014 (WK.3) | 50 | 2 | 4.3 | 13 |
| 1/30/2014 (WK. 5) | 50 | 52 | 3.5 | 12 |
| 2/13/2014 (WK. 7) | 50 | 52 | 3.5 | 12 |
| 2/27/2014 (WK. 9) | 50 | 52 | 3.5 | 9 |
| 3/13/2014 (WK. 11) | 50 | 52 | 3.5 | 12 |
| 3/27/2014 (WK. 13) | 50 | 52 | 3.5 | 12 |

B)

|  | **Start week^2^** | **Peak week^2^** | **Peak percentage** | **Duration of influenza season** |
| --- | --- | --- | --- | --- |
| HHS Region 5 | 50 | 52 | 3.6 | 15 |

**^1^**ILINet data are based on a reporting week that starts on Sunday and ends on Saturday of each week, and data are reported out through the FluView surveillance report the following Friday. Therefore, the most current ILINet data can lag the calendar date by 1–2 weeks.

**^2^**Weeks are given in Morbidity and Mortality Weekly Report surveillance weeks. For calendar start and end dates of each week, please see <http://wwwn.cdc.gov/nndss/script/downloads.aspx>.

Supplemental Table 6. A) Median predicted start week, peak week, peak % ILINet, and duration of season submitted by the 9 teams that completed the CDC Predict the 2013–2014 Influenza Season Challenge, by the date of submission, Health and Human Services Region 6.

B) The start week, peak week, peak ILINet percentage, and duration of season for the 2013–2014 influenza season as calculated from the U.S. Outpatient Influenza-like Illness Surveillance Network (ILINet), United States.

A)

| **Date of prediction**  **(Week of ILINet data availability^1,2^)** | **Median predicted start week^2^** | **Median predicted peak week^2^** | **Median predicted peak percentage** | **Median predicted duration of influenza season** |
| --- | --- | --- | --- | --- |
| 12/2/2013 (WK. 49) | 48 | 5 | 3.8 | 8 |
| 12/19/2013 (WK. 51) | 46 | 1 | 6.5 | 15 |
| 1/2/2014 (WK. 1) | 46 | 1 | 8.4 | 16 |
| 1/16/2014 (WK.3) | 45 | 52 | 9.8 | 16 |
| 1/30/2014 (WK. 5) | 45 | 52 | 9.8 | 16 |
| 2/13/2014 (WK. 7) | 45 | 52 | 9.9 | 15 |
| 2/27/2014 (WK. 9) | 45 | 52 | 9.9 | 15 |
| 3/13/2014 (WK. 11) | 45 | 52 | 9.9 | 17 |
| 3/27/2014 (WK. 13) | 45 | 52 | 9.9 | 19 |

B)

|  | **Start week^2^** | **Peak week^2^** | **Peak percentage** | **Duration of influenza season** |
| --- | --- | --- | --- | --- |
| HHS Region 6 | 45 | 52 | 9.9 | 20 |

**^1^**ILINet data are based on a reporting week that starts on Sunday and ends on Saturday of each week, and data are reported out through the FluView surveillance report the following Friday. Therefore, the most current ILINet data can lag the calendar date by 1–2 weeks.

**^2^**Weeks are given in Morbidity and Mortality Weekly Report surveillance weeks. For calendar start and end dates of each week, please see <http://wwwn.cdc.gov/nndss/script/downloads.aspx>.

Supplemental Table 7. A) Median predicted start week, peak week, peak % ILINet, and duration of season submitted by the 9 teams that completed the CDC Predict the 2013–2014 Influenza Season Challenge, by the date of submission, Health and Human Services Region 7.

B) The start week, peak week, peak ILINet percentage, and duration of season for the 2013–2014 influenza season as calculated from the U.S. Outpatient Influenza-like Illness Surveillance Network (ILINet), United States.

A)

| **Date of prediction**  **(Week of ILINet data availability^1,2^)** | **Median predicted start week^2^** | **Median predicted peak week^2^** | **Median predicted peak percentage** | **Median predicted duration of influenza season** |
| --- | --- | --- | --- | --- |
| 12/2/2013 (WK. 49) | 1 | 6 | 3.5 | 10 |
| 12/19/2013 (WK. 51) | 51 | 4 | 4.9 | 10 |
| 1/2/2014 (WK. 1) | 51 | 3 | 6.7 | 10 |
| 1/16/2014 (WK.3) | 51 | 2 | 5.2 | 9 |
| 1/30/2014 (WK. 5) | 51 | 52 | 4.5 | 9 |
| 2/13/2014 (WK. 7) | 51 | 52 | 4.5 | 9 |
| 2/27/2014 (WK. 9) | 51 | 52 | 4.5 | 8 |
| 3/13/2014 (WK. 11) | 51 | 52 | 4.5 | 8 |
| 3/27/2014 (WK. 13) | 51 | 52 | 4.5 | 8 |

B)

|  | **Start week^2^** | **Peak week^2^** | **Peak percentage** | **Duration of influenza season** |
| --- | --- | --- | --- | --- |
| HHS Region 7 | 51 | 52 | 4.5 | 8 |

**^1^**ILINet data are based on a reporting week that starts on Sunday and ends on Saturday of each week, and data are reported out through the FluView surveillance report the following Friday. Therefore, the most current ILINet data can lag the calendar date by 1–2 weeks.

**^2^**Weeks are given in Morbidity and Mortality Weekly Report surveillance weeks. For calendar start and end dates of each week, please see <http://wwwn.cdc.gov/nndss/script/downloads.aspx>.

Supplemental Table 8. A) Median predicted start week, peak week, peak % ILINet, and duration of season submitted by the 9 teams that completed the CDC Predict the 2013–2014 Influenza Season Challenge, by the date of submission, Health and Human Services Region 8.

B) The start week, peak week, peak ILINet percentage, and duration of season for the 2013–2014 influenza season as calculated from the U.S. Outpatient Influenza-like Illness Surveillance Network (ILINet), United States.

A)

| **Date of prediction**  **(Week of ILINet data availability^1,2^)** | **Median predicted start week^2^** | **Median predicted peak week^2^** | **Median predicted peak percentage** | **Median predicted duration of influenza season** |
| --- | --- | --- | --- | --- |
| 12/2/2013 (WK. 49) | 52 | 5 | 1.6 | 4 |
| 12/19/2013 (WK. 51) | 50 | 4 | 3.7 | 12 |
| 1/2/2014 (WK. 1) | 49 | 3 | 3.6 | 13 |
| 1/16/2014 (WK.3) | 49 | 2 | 3.8 | 13 |
| 1/30/2014 (WK. 5) | 49 | 52 | 3.7 | 11 |
| 2/13/2014 (WK. 7) | 49 | 52 | 3.7 | 11 |
| 2/27/2014 (WK. 9) | 49 | 52 | 3.7 | 11 |
| 3/13/2014 (WK. 11) | 49 | 52 | 3.7 | 12 |
| 3/27/2014 (WK. 13) | 49 | 52 | 3.7 | 12 |

B)

|  | **Start week^2^** | **Peak week^2^** | **Peak percentage** | **Duration of influenza season** |
| --- | --- | --- | --- | --- |
| HHS Region 8 | 49 | 52 | 3.7 | 12 |

**^1^**ILINet data are based on a reporting week that starts on Sunday and ends on Saturday of each week, and data are reported out through the FluView surveillance report the following Friday. Therefore, the most current ILINet data can lag the calendar date by 1–2 weeks.

**^2^**Weeks are given in Morbidity and Mortality Weekly Report surveillance weeks. For calendar start and end dates of each week, please see <http://wwwn.cdc.gov/nndss/script/downloads.aspx>.

Supplemental Table 9. A) Median predicted start week, peak week, peak % ILINet, and duration of season submitted by the 9 teams that completed the CDC Predict the 2013–2014 Influenza Season Challenge, by the date of submission, Health and Human Services Region 9.

B) The start week, peak week, peak ILINet percentage, and duration of season for the 2013–2014 influenza season as calculated from the U.S. Outpatient Influenza-like Illness Surveillance Network (ILINet), United States.

A)

| **Date of prediction**  **(Week of ILINet data availability^1,2^)** | **Median predicted start week^2^** | **Median predicted peak week^2^** | **Median predicted peak percentage** | **Median predicted duration of influenza season** |
| --- | --- | --- | --- | --- |
| 12/2/2013 (WK. 49) | 1 | 9 | 3.3 | 4 |
| 12/19/2013 (WK. 51) | 2 | 5 | 3.5 | 4 |
| 1/2/2014 (WK. 1) | 2 | 5 | 3.7 | 9 |
| 1/16/2014 (WK.3) | 51 | 2 | 5.1 | 13 |
| 1/30/2014 (WK. 5) | 51 | 1 | 4.9 | 13 |
| 2/13/2014 (WK. 7) | 51 | 2 | 4.7 | 12 |
| 2/27/2014 (WK. 9) | 51 | 4 | 5.0 | 10 |
| 3/13/2014 (WK. 11) | 51 | 4 | 4.9 | 10 |
| 3/27/2014 (WK. 13) | 51 | 4 | 4.9 | 11 |

B)

|  | **Start week^2^** | **Peak week^2^** | **Peak percentage** | **Duration of influenza season** |
| --- | --- | --- | --- | --- |
| HHS Region 9 | 51 | 4 | 4.7 | 10 |

**^1^**ILINet data are based on a reporting week that starts on Sunday and ends on Saturday of each week, and data are reported out through the FluView surveillance report the following Friday. Therefore, the most current ILINet data can lag the calendar date by 1–2 weeks.

**^2^**Weeks are given in Morbidity and Mortality Weekly Report surveillance weeks. For calendar start and end dates of each week, please see <http://wwwn.cdc.gov/nndss/script/downloads.aspx>.

Supplemental Table 10. A) Median predicted start week, peak week, peak % ILINet, and duration of season submitted by the 9 teams that completed the CDC Predict the 2013–2014 Influenza Season Challenge, by the date of submission, Health and Human Services Region 10.

B) The start week, peak week, peak ILINet percentage, and duration of season for the 2013–2014 influenza season as calculated from the U.S. Outpatient Influenza-like Illness Surveillance Network (ILINet), United States.

A)

| **Date of prediction**  **(Week of ILINet data availability^1,2^)** | **Median predicted start week^2^** | **Median predicted peak week^2^** | **Median predicted peak percentage** | **Median predicted duration of influenza season** |
| --- | --- | --- | --- | --- |
| 12/2/2013 (WK. 49) | 52 | 8 | 3.1 | 12 |
| 12/19/2013 (WK. 51) | 2 | 11 | 3.7 | 10 |
| 1/2/2014 (WK. 1) | 51 | 4 | 5.6 | 13 |
| 1/16/2014 (WK.3) | 51 | 3 | 5.1 | 16 |
| 1/30/2014 (WK. 5) | 51 | 1 | 4.2 | 15 |
| 2/13/2014 (WK. 7) | 51 | 1 | 4.1 | 13 |
| 2/27/2014 (WK. 9) | 51 | 1 | 4.0 | 8 |
| 3/13/2014 (WK. 11) | 51 | 1 | 4.0 | 8 |
| 3/27/2014 (WK. 13) | 51 | 1 | 4.0 | 8 |

B)

|  | **Start week^2^** | **Peak week^2^** | **Peak percentage** | **Duration of influenza season** |
| --- | --- | --- | --- | --- |
| HHS Region 10 | 51 | 1 | 4.0 | 8 |

**^1^**ILINet data are based on a reporting week that starts on Sunday and ends on Saturday of each week, and data are reported out through the FluView surveillance report the following Friday. Therefore, the most current ILINet data can lag the calendar date by 1–2 weeks.

**^2^**Weeks are given in Morbidity and Mortality Weekly Report surveillance weeks. For calendar start and end dates of each week, please see <http://wwwn.cdc.gov/nndss/script/downloads.aspx>.

Supplemental Table 11. Forecasts that were within 1 week of the start week of the season, as calculated from ILINet during the 2013–14 influenza season, by the 9 teams that forecasted all required milestones for the CDC Predict the Influenza Season Challenge, by the date of submission, United States (N=13 forecasts).

| **Date of forecast**  **(Week of ILINet data availability^1,2^)** | **Team and Forecast** | | | | | | | | | | | | |
| --- | --- | --- | --- | --- | --- | --- | --- | --- | --- | --- | --- | --- | --- |
|  | **A** | **B** | **C1** | **C2** | **C3** | **C4** | **D1** | **D2** | **E** | **F** | **G** | **H** | **I** |
| 12/2/2013 (WK. 46) | Y | N | N | N | N | N | N | N | N | N | Y | Y | N |
| 12/19/2013 (WK. 49) | N | Y | Y | N | Y | N | Y | N | N | Y | Y | N | N |
| 1/2/2014 (WK. 51) | Y | Y | Y | Y | Y | Y | Y | Y | N | Y | Y | Y | Y |
| 1/16/2014 (WK.1) | Y | Y | Y | Y | Y | Y | Y | Y | N | Y | Y | Y | Y |
| 1/30/2014 (WK. 3) | Y | Y | Y | Y | Y | Y | Y | Y | N | Y | Y | Y | N |
| 2/13/2014 (WK. 5) | Y | Y | Y | Y | Y | Y | Y | Y | N | Y | Y | Y | N |
| 2/27/2014 (WK. 7) | Y | Y | Y | Y | Y | Y | Y | Y | N | Y | Y | Y | N |
| 3/13/2014 (WK. 9) | Y | Y | Y | Y | Y | Y | Y | Y | N | Y | Y | Y | N |
| 3/27/2014 (WK. 11) | N | Y | Y | Y | Y | Y | Y | Y | N | Y | Y | Y | N |

Supplemental Table 12. Forecasts that were within 1 week of the peak week of the season, as calculated from ILINet during the 2013–14 influenza season, by the 9 teams that forecasted all required milestones for the CDC Predict the Influenza Season Challenge, by the date of submission, United States (N=13 forecasts).

| **Date of forecast**  **(Week of ILINet data availability^1,2^)** | **Team and Forecast** | | | | | | | | | | | | |
| --- | --- | --- | --- | --- | --- | --- | --- | --- | --- | --- | --- | --- | --- |
|  | **A** | **B** | **C1** | **C2** | **C3** | **C4** | **D1** | **D2** | **E** | **F** | **G** | **H** | **I** |
| 12/2/2013 (WK. 46) | N | N | N | N | N | N | N | N | N | N | Y | N | N |
| 12/19/2013 (WK. 49) | N | N | N | N | N | N | N | N | Y | N | N | N | Y |
| 1/2/2014 (WK. 51) | N | N | N | Y | N | N | N | N | Y | N | N | N | N |
| 1/16/2014 (WK.1) | N | Y | Y | N | Y | N | N | N | Y | Y | N | Y | N |
| 1/30/2014 (WK. 3) | N | Y | Y | Y | Y | Y | Y | N | Y | Y | Y | Y | Y |
| 2/13/2014 (WK. 5) | N | Y | Y | Y | Y | Y | Y | N | N | Y | Y | Y | Y |
| 2/27/2014 (WK. 7) | N | Y | Y | Y | Y | Y | Y | Y | N | Y | Y | Y | Y |
| 3/13/2014 (WK. 9) | N | Y | Y | Y | Y | Y | Y | Y | N | Y | Y | Y | Y |
| 3/27/2014 (WK. 11) | N | Y | Y | Y | Y | Y | Y | Y | Y | Y | Y | Y | Y |

Supplemental Table 13. Forecasts that were within 1 percent of the peak ILINet percentage, as calculated from ILINet during the 2013–14 influenza season, by the 9 teams that forecasted all required milestones for the CDC Predict the Influenza Season Challenge, by the date of submission, United States (N=13 forecasts).

| **Date of forecast**  **(Week of ILINet data availability^1,2^)** | **Team and Forecast** | | | | | | | | | | | | |
| --- | --- | --- | --- | --- | --- | --- | --- | --- | --- | --- | --- | --- | --- |
|  | **A** | **B** | **C1** | **C2** | **C3** | **C4** | **D1** | **D2** | **E** | **F** | **G** | **H** | **I** |
| 12/2/2013 (WK. 46) | N | N | Y | N | N | N | N | N | N | N | Y | N | Y |
| 12/19/2013 (WK. 49) | Y | N | N | N | N | Y | Y | Y | N | N | Y | N | Y |
| 1/2/2014 (WK. 51) | Y | N | N | Y | N | Y | N | Y | N | N | Y | N | N |
| 1/16/2014 (WK.1) | Y | Y | Y | N | Y | N | Y | Y | N | Y | Y | Y | Y |
| 1/30/2014 (WK. 3) | Y | Y | Y | Y | Y | Y | Y | Y | N | Y | Y | Y | N |
| 2/13/2014 (WK. 5) | Y | Y | Y | Y | Y | Y | Y | Y | Y | Y | Y | Y | N |
| 2/27/2014 (WK. 7) | Y | Y | Y | Y | Y | Y | Y | Y | Y | Y | Y | Y | Y |
| 3/13/2014 (WK. 9) | Y | Y | Y | Y | Y | Y | Y | Y | Y | Y | Y | Y | Y |
| 3/27/2014 (WK. 11) | Y | Y | Y | Y | Y | Y | Y | Y | Y | Y | Y | Y | Y |

Supplemental Table 14. Forecasts that were within 1 week of the duration of the season, as calculated from ILINet during the 2013–14 influenza season, by the 9 teams that forecasted all required milestones for the CDC Predict the Influenza Season Challenge, by the date of submission, United States (N=13 forecasts).

| **Date of forecast**  **(Week of ILINet data availability^1,2^)** | **Team and Forecast** | | | | | | | | | | | | |
| --- | --- | --- | --- | --- | --- | --- | --- | --- | --- | --- | --- | --- | --- |
|  | **A** | **B** | **C1** | **C2** | **C3** | **C4** | **D1** | **D2** | **E** | **F** | **G** | **H** | **I** |
| 12/2/2013 (WK. 46) | N | Y | N | N | N | N | Y | Y | N | N | N | Y | N |
| 12/19/2013 (WK. 49) | N | N | Y | N | Y | Y | Y | Y | Y | N | N | N | N |
| 1/2/2014 (WK. 51) | N | Y | Y | Y | N | Y | Y | N | Y | N | Y | N | N |
| 1/16/2014 (WK.1) | N | N | Y | Y | N | N | Y | Y | Y | N | Y | N | N |
| 1/30/2014 (WK. 3) | N | N | Y | Y | N | N | Y | Y | Y | N | Y | N | N |
| 2/13/2014 (WK. 5) | N | N | Y | N | N | Y | N | Y | Y | Y | Y | N | N |
| 2/27/2014 (WK. 7) | N | N | N | N | Y | N | N | Y | Y | Y | Y | N | N |
| 3/13/2014 (WK. 9) | N | N | Y | Y | Y | Y | Y | Y | Y | Y | Y | N | N |
| 3/27/2014 (WK. 11) | N | Y | Y | Y | Y | Y | Y | Y | N | Y | Y | Y | N |
